# Object location memories recruit distal CA1 and catecholaminergic inputs to proximodistal CA1

**DOI:** 10.1371/journal.pone.0337834

**Published:** 2025-12-04

**Authors:** Ana Paula Crestani, Yusuke Teratani-Ota, Brian Joseph Wiltgen

**Affiliations:** 1 Department of Psychology, University of California, Davis, California, United States of America; 2 Center for Neuroscience, University of California, Davis, California, United States of America; University of Nebraska Medical Center College of Medicine, UNITED STATES OF AMERICA

## Abstract

The hippocampus is thought to combine “what” and “where” information from the cortex so that objects and events can be represented within the spatial context in which they occur. Surprisingly then, these distinct types of information remain partially segregated in the output region of the hippocampus, area CA1. In this region, objects preferentially activate neurons in the distal segment (adjacent to the subiculum) while spatial locations are precisely represented by neurons in the proximal segment (adjacent to CA2). This difference likely results from distinct anatomical connections; proximal CA1 receives direct input from the medial entorhinal cortex (which encodes spatial context) whereas distal CA1 has reciprocal connections with the lateral entorhinal cortex (which encodes objects and events). Based on these findings, it has been proposed that CA1 contains two distinct representations; one that encodes the animal’s spatial location and another that encodes objects that are present in the environment. The current study aimed to determine the role of distal CA1 in learning the location of objects in an environment. To do this, we first demonstrated that distal CA1 is more responsive (higher levels of c-Fos) to objects while proximal is spatially tuned. Further, as previous studies indicate that catecholamines can regulate CA1 activity, we lesioned the catecholaminergic inputs to CA1 and observed a reduction in c-Fos levels in both segments of CA1, and an impairment in object location memory 24h after training. Together, these findings indicate that processing object location in an environment recruits distal CA1 and catecholaminergic inputs to CA1.

## Introduction

The hippocampus (HPC) is thought to form episodic memories by integrating spatial and non-spatial inputs from the entorhinal cortex (EC). However, the way these distinct types of information are associated in different subregions of the HPC is not completely understood. Since the discovery of place cells, animal work has traditionally focused on hippocampal contributions to spatial learning and memory [1,2]. However, we now know that the HPC processes many different types of information. For example, time cells fire in precise temporal sequences, which is thought to allow spatial and non-spatial information to be linked across time [3–5]. Recent studies have also demonstrated that CA1 pyramidal neurons respond to specific auditory cues, odors, and objects [6–9]. These responses reflect the diverse types of information the HPC receives from cortical and subcortical areas.

The entorhinal cortex (EC) is the major cortical input to the HPC. It can be subdivided into the medial entorhinal cortex (MEC) and the lateral entorhinal cortex (LEC), which are functionally distinct from each other. The MEC is important for forming allocentric representations of space via cells that fire in a grid-like pattern throughout the environment [10,11]. In addition to grid cells, the MEC contains border cells, speed cells, and head-direction cells, all of which are important for spatial navigation [12–14]. Cells in LEC are not spatially modulated but instead responsive to objects or odors in the environment. For instance, LEC neurons fire near objects that are encountered during an open foraging task [15]. The removal of an object causes a different set of cells to fire at the site where it was previously located. This suggests the LEC stores and retrieves associations between objects and their spatial location [16]. Interestingly, LEC neurons show little to no firing in response to visual landmarks that make up the spatial environment [17]. Instead, this information is processed by the MEC and used to form allocentric representations of the environment that are necessary for spatial navigation.

The anatomical connections between the EC and the HPC have been characterized extensively. The MEC and LEC both send direct projections to granule cells in the dentate gyrus via the perforant path. Cells in DG transmit this information to pyramidal neurons in CA3 via the mossy fiber pathway. And finally, Schaffer collaterals from CA3 neurons terminate on cells in CA1. CA1 neurons also receive a direct projection from the EC. This projection, called the temporoammonic pathway, bypasses DG and CA3 and synapses directly on CA1 neurons. Interestingly, the inputs from MEC and LEC are segregated along the proximodistal axis of CA1. This is not the case in DG and CA3, where individual neurons receive input from both regions. The proximal segment of CA1 (adjacent to CA2) receives input from the MEC while the distal segment (adjacent to the subiculum) is targeted by neurons in the LEC [18,19]. These segments then send return projections back to the deep layers of EC; proximal CA1 to MEC and distal CA1 to LEC. Based on this anatomy, it is hypothesized that the proximal CA1 determines the animal’s position in space, while distal CA1 encodes non-spatial stimuli in the environment, like objects and odors.

Consistent with this idea, *in vivo* electrophysiology recordings and immediate early genes studies have shown that distal CA1 responds more strongly to the presence of objects than proximal CA1. In addition, the place cells in proximal CA1 (which respond to the animal’s location) are more precise and stable than those in distal CA1 [7,20]. Our lab showed that excitotoxic lesions of proximal, but not distal CA1, make it difficult for animals’ to recognize a familiar spatial environment [21].

Pyramidal cell activity is strongly modulated by catecholamines released from locus coeruleus (LC) and ventral tegmental area (VTA) terminals in CA1. While the VTA is known to release dopamine in many brain areas, the LC co-releases norepinephrine and dopamine in the HPC [22]. In fact, catecholaminergic inputs to the HPC primarily come from LC neurons [23–25]. Most of the VTA projections to the HPC are GABAergic or glutamatergic and only 10% are dopaminergic. Although small in number, these inputs have been shown to stabilize hippocampal representations of space [26]. Catecholamine release in the HPC modulate entorhinal inputs in a bidirectional and frequency dependent manner. During low-frequency stimulation, dopamine attenuates the direct entorhinal input to CA1 [27,28]. In contrary, under high-frequency stimulation protocols, dopamine potentiates entorhinal inputs in CA1. These effects have been observed in both proximal and distal CA1 [29]. Unlike dopamine, norepinephrine primarily modulates entorhinal inputs to proximal CA1. This implies that dopamine and norepinephrine play distinct roles in filtering the transmission of sensory information from the entorhinal cortex to proximal and distal CA1.

Despite these data, it is still not known if activity in distal CA1 is required to learn and remember the position of objects in space. The majority of studies to date have simply recorded single unit activity in distal CA1 while animals are exposed to objects. However, this activity has not been directly linked to memory performance [7,30–32]. In our study, we aimed to determine the role of distal CA1 in learning the location of objects in an environment. We started by using c-Fos expression as a proxy to assess whether neuronal activity in distinct segments of CA1 was modulated by the stimuli to which the animals were exposed (object vs. novel environment). We then investigated whether catecholamines are responsible for the object induced c-Fos expression. Finally, we explored the role of catecholamines on learning object locations using a neurotoxin 6-OHDA to lesion catecholaminergic input to the dorsal HPC.

## Results

### Distal CA1 is more responsive to objects while proximal CA1 is spatially tuned

**Fig 1:**
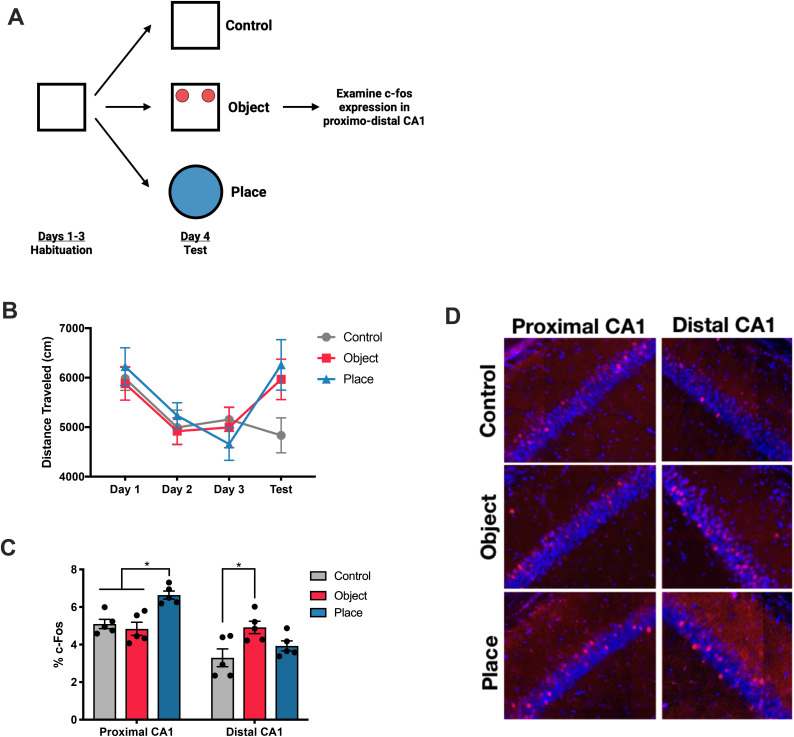
Proximal and distal CA1 are modulated by different stimuli. **(A)** Experimental design. Animals were habituated to an open-field environment for fifteen minutes per day for three days. Following habituation, animals were placed in the habituated open-field environment (control), exposed to novel objects (object), or put into a novel place (place) (n = 5 per group). **(B)** All groups habituated to the environment across habituation. Activity levels increased when mice were exposed to novel objects or a new place (2-way repeated measures ANOVA, Interaction F(6, 36) = 3.707, p < 0.01). **(C)** Quantification of c-Fos expression in proximal and distal CA1. Mice exposed to a new place displayed higher levels of c-Fos in proximal CA1 while mice exposed to novel objects had higher levels of c-Fos in distal CA1 (2-way repeated ANOVA, Interaction F(2, 12) = 12.04, p < 0.01, Sidak’s multiple comparisons test p < 0.01). **(D)** Representative images of c-Fos expression in proximal and distal CA1 from mice in each condition.

Previous studies found proximal and distal CA1 responsive to spatial and non-spatial stimuli, respectively [11,20,33]. To replicate previous findings, we habituated animals to an open-field environment for three consecutive days. Following habituation, animals were either placed in a habituated environment (control), exposed to a pair of identical objects in the habituated environment (object), or placed in a novel environment (place) **(Fig 1A)**. After the test session, we examined c-Fos as a proxy for neural activity along the proximodistal axis of CA1 to determine what types of stimuli each segment processes.

As expected, animals in all groups showed a gradual decrease in the amount of distance traveled in the environment during habituation **(Fig 1B**; **Days 1–3)**. Although there was a significant interaction of days and condition (2-way repeated measures ANOVA, Interaction F(6, 36) = 3.707, p < 0.01), post-hoc tests did not reveal any significant differences. However, it is still worthwhile to observe that animals exposed to objects or a novel place on the test day displayed increased locomotor activity levels. Quantification of c-Fos expression revealed elevated levels in proximal CA1 of mice that were exposed to a novel place compared to control mice and those exposed to objects (2-way repeated measures ANOVA, Interaction Segment x Condition F(2, 12) = 12.04, p < 0.01, Sidak’s multiple comparisons test p < 0.01). We also observed elevated c-Fos activity in distal CA1 of mice exposed to a novel object compared to mice exposed to the control condition (Sidak’s multiple comparisons test p < 0.01) **(Figs 1C** **and** **1D)**. Despite animals in the object and place conditions displaying similar locomotor activity levels during testing, we observed differential activity patterns along the proximo-distal axis of CA1. Thus, our observation cannot simply be due to animals being more active in their assigned conditions. These results replicate previous studies suggesting that proximal CA1 is more spatially tuned while distal CA1 is responsive to objects.

### Catecholaminergic lesions reduces c-Fos levels in both proximodistal axis of CA1

**Fig 2:**
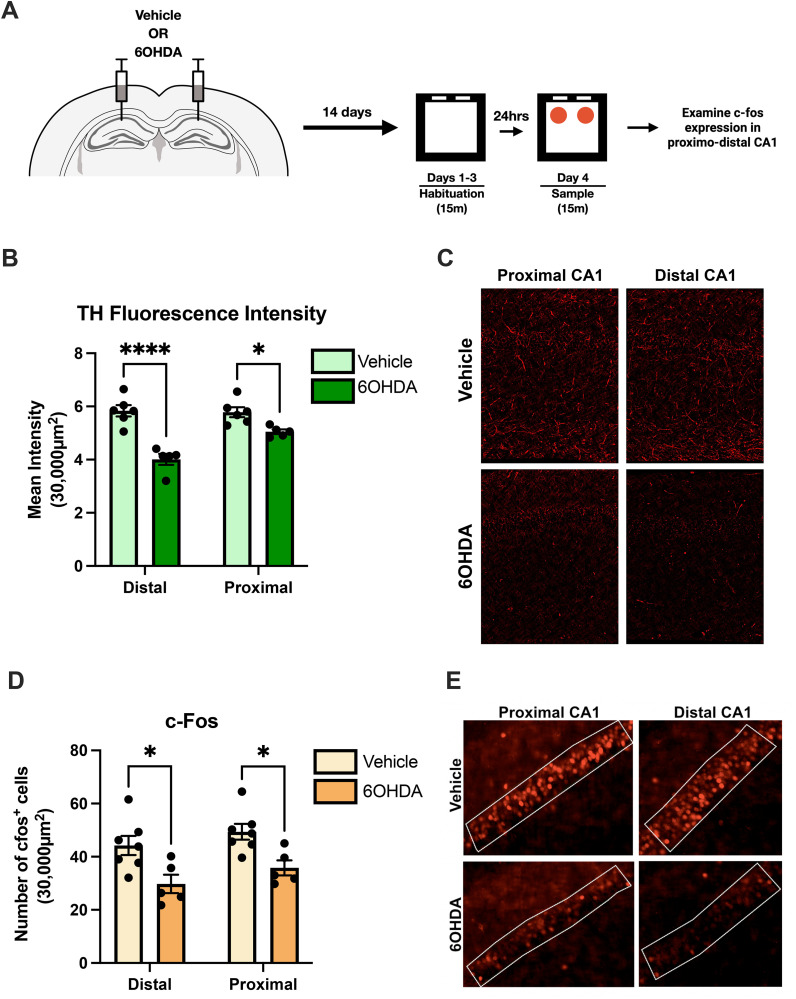
Distal 6-OHDA catecholaminergic lesions reduced c-Fos expression in proximal and distal CA1. **(A)** Experimental design. Animals received bilateral infusions of 6-OHDA or saline in the HPC. After recovery, animals habituated to an open-field environment for three consecutive days (fifteen min per day). The following day, both groups of animals were exposed to novel objects. Ninety-minutes after, animals were sacrificed, and brain tissue was fixed to immunostain for c-Fos. **(B)** Quantification of tyrosine-hydroxylase (TH) in HPC. A reduction of TH-positive axons in distal and proximal CA1 were observed in the 6-OHDA animals (n = 5) compared to control group (n = 6) (2-way repeated measures ANOVA, Interaction F(1, 9) = 24.60, p < 0.001, Sidak’s multiple comparisons test, p < 0.0001 for distal and p < 0.05 for proximal). **(C)** Representative images of TH expression in proximal and distal CA1. **(D)** Quantification of c-Fos expression in proximal and distal CA1. Catecholaminergic lesions with 6-OHDA reduced overall c-Fos expression (n = 5) in proximal and distal CA1 compared to control group (n = 6) (2-way repeated measures ANOVA, main effect of lesion: F(1, 9) = 8.761, p < 0.05; main effect of segment: F(1, 9) = 91.91, p < 0.0001). **(E)** Representative images of c-Fos expression in proximal and distal CA1 of 6-OHDA and vehicle animals.

Catecholamines have been shown to be important in regulating entorhinal inputs along the transverse axis of CA1 [27,29,32]. We next investigated whether catecholamines are responsible for the object induced c-Fos expression we observed in our previous experiment. To do this, a neurotoxin 6-hydroxydopamine (6-OHDA) hydrobromide was bilaterally infused into the HPC to lesion all catecholaminergic inputs. After recovery, animals were placed in an open-field environment for three consecutive days and then exposed to a pair of identical objects during the sample session **(Fig 2A)**. We then performed immunohistochemistry to examine c-Fos activity in proximodistal CA1 following the behavioral procedure. To confirm 6-OHDA lesions, we quantified tyrosine hydroxylase (TH) expression in proximal and distal CA1 via immunohistochemistry. TH is an enzyme that catalyzes the first step in the biosynthesis of catecholamines, converting tyrosine to L-DOPA. Animals with 6-OHDA lesions had significantly lower TH-positive axons in the HPC in both segments of CA1 relative to the vehicle condition (2-way repeated measures ANOVA, Interaction F(1, 9) = 24.60, p < 0.001, Sidak’s multiple comparisons test, p < 0.0001 for distal and p < 0.05 for proximal) **(Figs 2B** **and** **2C)**. In addition, analysis of c-Fos expression revealed that the lesion caused a reduction in c-Fos levels in both segments of CA1 (2-way repeated measures ANOVA, main effect of lesion: F(1, 9) = 8.761, p < 0.05; main effect of segment: F(1, 9) = 91.91, p < 0.0001) **(Figs 2D** **and** **2E)**. These results suggest that catecholamines may be important for increasing c-Fos expression driven by object exploration but does not differentially modulate activity along the proximodistal axis of CA1.

### Catecholaminergic lesions impair object location memory

**Fig 3:**
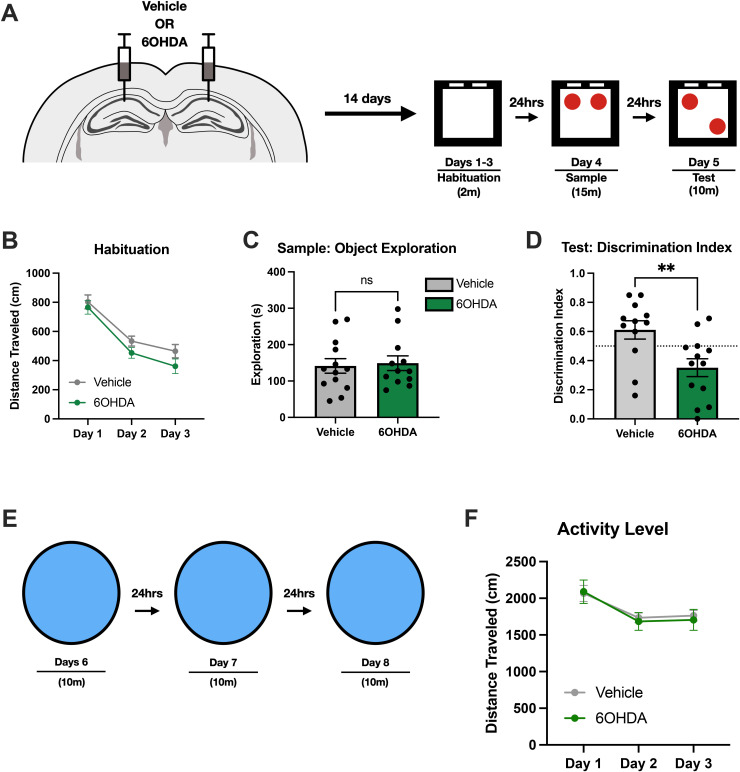
Catecholaminergic inputs are required for appropriate object discrimination. **(A)** Experimental Design of OLM. 6-OHDA (n = 13) or saline (n = 12) was bilaterally infused in the HPC and performed the OLM task following recovery. **(B)** Both groups habituated to the environment across the days. Activity levels between both groups were similar throughout habituation (2-way repeated measures ANOVA, Interaction F(2, 46) = 1.198, p > 0.05). **(C)** Total object exploration during sample session. No differences were observed between the 6-OHDA and vehicle group (t(23) =0.2705, p > 0.05). **(D)** Performance of OLM measured by discrimination index at testing. Animals with 6-OHDA lesions had impaired discrimination relative to the vehicle group (t(23) = 2.958, p < 0.05). **(E)** Experimental design of habituation task. The same animals were exposed to a novel place for three consecutive days, fifteen-minutes per day. **(F)** Both groups habituated to the novel place in a similar manner (2-way repeated measures ANOVA, Interaction F(2, 46) = 0.4278, p > 0.05).

To examine the contribution of catecholamines on object-related memories, mice with catecholaminergic lesions performed the hippocampal-dependent object location memory (OLM) task. In this task, animals habituated to an open-field environment for three consecutive days. The following day, animals were presented with a pair of identical objects in the habituated environment. After a 24-hour delay, one of the presented objects was moved to a new location and animals were given time to explore **(Fig 3A)**. As mice are naturally inclined to explore novelty, they should spend more time exploring the object that moved to a new location. Although LC inputs in the HPC have been shown to be important for learning new contexts [25], we found no differences in activity levels during habituation to the open-field environment (2-way repeated measures ANOVA, Interaction F(2, 46) = 1.198, p > 0.05) **(Fig 3B)**. At testing, mice with 6OHDA lesions had impaired discrimination when compared with the vehicle group (t(23) = 2.958, p < 0.01) **(Fig 3D)**. The deficit cannot be explained by differences in exploration behavior during the sample session as both groups explored objects in a similar manner (t(23) =0.2705, p > 0.05) **(Fig 3C)**.

Deficits may not have been observed during the habituation sessions from the OLM task as animals were only given a short time frame (2 minutes) to explore the environment. Differences may have been observed if animals were given a longer period to explore. The same animals from the OLM experiment performed a habituation task in a novel environment; animals were allowed 10 minutes to explore a novel environment for three consecutive days **(Fig 3G)**. No differences were observed in the activity levels across days in both groups, suggesting that catecholaminergic inputs are not required for habituating to new contexts (2-way repeated measures ANOVA, F(2, 46) = 0.4278, p > 0.05) **(Fig 3F)**. Due to 6OHDA lesions being a chronic manipulation, there is sufficient time for changes in the brain to occur to compensate for the lack of catecholaminergic inputs in the HPC. However, this compensation is not sufficient to allow animals to properly learn object-related information for the OLM task. As 6-OHDA lesions prevent the release of both dopamine and norepinephrine, we cannot make any conclusions about which catecholamine is important for processing object-related information.

In summary, the results of this experiment show that the absence of catecholaminergic input to CA1 does not impair habituation **(Figs 3B** **and** **Fig 3F)** or object exploration time during the sample session **(Fig 3C)**. However, lesioned animals exhibit impaired object location memory during the test session **(Fig 3D)**.

## Discussion

Here, we observed differential activity patterns along the proximodistal axis of CA1. Proximal CA1 showed an increase in c-Fos activity after exposure to a novel place. In the contrary, animals exposed to objects had elevated levels of c-Fos in distal CA1. This result is consistent with previous findings suggesting that proximal CA1 is spatially tuned while distal CA1 is responsive to objects. Despite the overall activity between the object and place conditions being similar, we observed distinct c-Fos expression at each segment of CA1. Thus, the pattern we observed is driven by the type of stimuli instead of increased activity levels.

We also found that catecholaminergic lesions in the HPC greatly reduced c-Fos activity in proximal and distal CA1 following exposure to objects. Furthermore, animals without catecholaminergic inputs had impaired discrimination during the testing of the OLM task. Our results are consistent with previous studies suggesting that catecholamines are important for object-related memory tasks [23,34]. Surprisingly, our manipulation did not affect animals’ ability to learn about new contexts. Wagatsuma et al. had previously demonstrated that LC projections to CA3, but not CA1 or DG, were necessary for acquisition of a contextual memory [25]. In our study, catecholaminergic lesions were mostly targeted to the CA1 subregion of the hippocampus and thus we may not have observed a deficit in the animal’s contextual memory. Catecholamines in the CA1 may not be necessary for the encoding of new places; however, release of catecholamines can result in the enhancement of memories by modulating consolidation processes [23,35–37]. Future experiments using optogenetic tools that allow bidirectional control (inhibition and stimulation) of neuronal activity during acquisition, consolidation, and retrieval can elucidate in which mnemonic processes catecholamine release in CA1 is essential.

From our study, no conclusions can be made about whether dopamine is important for object-related memories. However, some studies provide evidence that dopamine may be the key neuromodulator for allowing animals to learn the OLM task. Kempadoo et al. (2016) optogenetically stimulated the LC in mice that received an intracranial infusion of SCH23390 (dopamine D1/D5 receptor antagonist) or propranolol, a beta-adrenergic receptor antagonist, prior to the sample session of a weak training protocol. Under the weak training protocol, animals had poor discrimination, but this was mitigated by optogenetically stimulating the LC. The enhancement in performance was blocked with SCH23390 but not propranolol, implicating that norepinephrine may not be necessary for learning about the location of objects. Although norepinephrine may not be involved in the acquisition of an object memory, *in vivo* microdialysis of the HPC demonstrated that there is a 2-to-3-fold increase in dopamine and norepinephrine release when an object was displaced during testing of the OLM task [34]. Therefore, norepinephrine may have a crucial role in the retrieval of an object-related memory or in detecting changes in the environment. The involvement of LC under normal learning conditions is still an open question. As catecholamines regulate the information flow of entorhinal inputs in CA1, it is important to understand how LC supports learning about an object’s location in the environment. In future experiments, we intend to manipulate subpopulations of LC neurons that may be involved in the animal’s exploration behavior.

As stated before, proximal and distal CA1 are typically thought to process spatial and non-spatial information, but this may be an oversimplification of the role of these two circuits within the HPC. While grid cells in MEC maintain their grid-like firing properties regardless of whether objects are present, it has been reported that there is a different type of cells called an object-vector cell that fires when an animal is facing an object at a specific orientation and a certain distance away from an object [38]. The firing properties of object vector cells remain the same even if the environment, the object, or the object’s location is different. Therefore, it is a purely allocentric representation of the animal’s position relative to an object in any environment. This representation allows the animal to navigate using landmarks to reach a goal destination. Object-vector cells have yet to be found in proximal CA1 but given that it mostly receives projections from the MEC, there are likely cells that show stable firing when an animal is at a certain distance from an object.

A recent study observed place cell activity in proximodistal CA1 when animals explored objects during the OLM task [33]. In addition to place cells, trace cells or cells that fire to an object’s previous location were also found in proximodistal CA1. Although object-related activity was found in both segments, cells in distal CA1, but not proximal, exhibited higher spatial specificity and were strongly modulated by the proximity of objects. For instance, trace cell activity of distal CA1 fired precisely to the displaced object’s previous location than proximal CA1. Instead of firing precisely to the object’s previous location, trace cell activity in proximal CA1 may be influenced by the animal’s head-direction and the animal’s distance from the object like object vector cells. These studies may explain why exposure to objects elicited similar levels of c-Fos expression in proximal and distal CA1. Proximal CA1 processes the precise location of an animal in relation to the object’s location, while distal CA1 encodes the location of objects in an environment, which is consistent with Knierim’s new framework of the parallel circuits within the hippocampal system [39]. The new framework describes the MEC-proximal CA1 circuit as being imperative for storing a spatial framework of a context, allowing animals to determine where they are in the environment. Animals may use distal cues or objects/landmarks to navigate to a goal of interest. In contrast, the LEC-distal CA1 circuit is important for storing information about the content of an experience. This can consist of individual items in the environment, what certain objects are, and the location of objects in the environment.

Due to the diverse responses in the HPC, future studies will need to use techniques such as miniscope calcium imaging to record the activity of individual neurons during the OLM task in order to better understand how proximodistal CA1 is involved in object-related memories. In addition, it will be of interest to utilize catecholaminergic biosensors (such as Dlight, GRAB_DA_, or GRAB_NE_) with calcium sensors to examine how dopamine or norepinephrine influence the activity of cells that respond to objects in each segment.

## Materials and methods

### Subjects

All procedures were approved by the Animal Care and Use Committee at UC Davis (Protocol #23233). For the first experiment, C57BL6 males were used from in-house bred mice. All other experiments used F1 male hybrids (C57BL/6N x 129S6; Taconic). Animals were all group-housed (3–4 animals by cage) prior to all experiments. Mice were maintained on a 12/12 light/dark cycle and were given free access to food and water. All surgeries were performed at 8 weeks of age and behavioral experiments began at 10–12 weeks of age. For experiments not requiring surgeries, behavioral experiments began at 8–12 weeks of age.

The animals’ health and behavior were monitored daily. Animals undergoing stereotaxic surgery were treated with analgesic (buprenorphine, 0.1 mg/kg) for the following three days to reduce any discomfort. After completing the experiments, the euthanasia method used in all experiments was perfusion under isoflurane anesthesia (5% for induction). A humane endpoint was planned to be applied if a significant weight loss (> 20% or more) and/or clinical signs of distress (e.g., lethargy, hunched postured, ruffled fur, labored breathing, pain) or severe suffering were observed. If animals met endpoint criteria, no more than 24 hours would elapse before euthanasia. No animals used in this study needed to be subject to humane endpoint or died before meeting criteria for euthanasia.

Regarding the duration of the experiments, all animals were handled for seven days before the start of behavioral experiments. The first experiment (Fig 1, n = 15) lasted 4 days. In the two following experiments, the animals underwent surgery and, two weeks later, the behavioral protocol, which lasted four days (Fig 2, n = 11) and eight days (Fig 3, n = 25), respectively.

### Behavioral apparatus

An open-field box (width: 37.5 x length: 37.5 x height:37.5 cm) made from white king starboard was used for the task. One of the walls was labeled with two vertical black stripes to serve as a spatial cue for the object-location memory task. An air purifier was turned on high to produce white noise in the room. Additionally, red lights were used for all behavioral experiments. For a novel context, a white PVC tube (diameter:37.5 x height:37.5 cm) was used with an air purifier was turned on low. Dim light settings were used to differentiate the context from the object-location memory task context.

### Novel object location memory task

Mice were placed into an open-field box for three-consecutive days for two-minutes each session. One of the walls was labeled with two vertical black stripes to serve as a spatial cue. During the sample session, mice were placed into the open-field box with a pair of identical objects located in front of the spatial cue for fifteen minutes. A stack (heigh: 14 cm) of 5 square blocks (width: 3 x length: 3 cm) was used as object. The apparatus was cleaned with 70% ethanol between animals. After a twenty-four-hour A stack (heigh: 14 cm) of 5 square blocks (width: 3 x leng: 3 cm) was used as object. delay, one of the two objects was moved to the opposite wall to test the animal’s memory for ten-minutes. The placement of objects was counterbalanced. The Noldus EthoVision Software was used for animal tracking and to quantify object exploration. Exploration was considered as the time the animal spent with its nose towards the object and within 2 centimeters of the object. The discrimination index was calculated by dividing the exploration for the novel object by the total exploration of objects (stationary and displaced). A discrimination index of 0.5 indicated performance at chance level. Due to the nature of the task, only the first five minutes was used to calculate the discrimination index.

### Stereotaxic surgeries

For lesion experiments, mice were anesthetized with isoflurane (5% for induction and 1,5% for maintenance), placed in the stereotaxic and then a craniotomy was performed with a small precision drill to deliver the solution with a glass pipette needle at an injection rate of 2nL per sec using a microinfusion pump (World Precision Instruments). Animals were bilaterally infused with 150 μL of either sterile saline or 6-Hydroxydopamine (6-OHDA) hydrobromide (HelloBio, HB1889) in the HPC. The coordinates to target CA1 were ([AP] –2.0 mm, [ML] ± 1.5 mm, [DV] −1.25 mm).

### c-Fos experiments

Animals were all single-housed prior to the start of a behavioral experiment to prevent any factors that may induce activity unrelated to the task. Before the start of behavioral experiments, all animals were handled for seven days (5-minutes each). For the first experiment, mice were habituated to the open-field environment for three consecutive days (15-minutes each) and then randomly assigned to one of three conditions: re-exposure to open field box (Control), exposure to novel objects in the open field box (Object), or exposure to a new context (Place). For lesion experiment, mice were randomly assigned to receive 6-OHDA or sham lesions. Following recovery, mice underwent the same behavioral procedure as the first experiment except they only underwent the object condition. Animals in both experiments were transcardially perfused ninety minutes from the start of the testing session and brains were fixed for 24 hour in preparation for immunohistochemistry analysis. The amount of distance traveled for each session was measured using Noldus Ethovision tracking software.

### Immunohistochemistry for c-Fos

40uM coronal sections were collected and stained for c-Fos. Prior to all antibody staining, slices were washed with 1X phosphate buffer solution (PBS) three times. Slices were incubated in donkey blocking buffer containing 2% donkey serum, 0.2% Triton X, and PBS for 15 minutes. Then, slices were incubated in c-Fos rabbit antibody for 24 hours at room temperature. A polyclonal rabbit antibody (1: 1000, Santa Cruz) was used for experiment 1 while a monoclonal rabbit antibody (1:5000, Cell signaling) was used for experiment 4. Following the primary antibody staining, slices were placed in biotinylated donkey anti-rabbit secondary antibody (1:500, Jackson ImmunoReseach) for sixty minutes at room temperature, followed by streptavidin-Cy3 (1:500, Jackson ImmunoResearch) for forty-five minutes). Finally, slices were DAPI stained for fifteen minutes and mounted on slides.

### Immunohistochemistry for TH

Separate 40um coronal slices from the same animals were collected to stain for tyrosine hydroxylase (TH). Prior to all antibody staining, coronal slices were washed with 1X phosphate buffer solution (PBS) three times. Slices were incubated in donkey blocking buffer. Next, slices were incubated in TH polyclonal rabbit antibody for 24 hours at room temperature. The following steps after the incubation of primary antibody is the same procedure as described in the c-Fos immunohistochemistry protocol.

### Imaging and quantification

Samples were imaged using an Olympus fluorescence virtual slide scanning microscope. For the first experiment, z-stack images were obtained for 40um slices. Fluorescent images were then imported into FIJI as a grayscale image. Each image’s channel was separated to hand count c-Fos positive cells through z-stacks. A 3D Objects Counter macro in FIJI was used to estimate the number of DAPI stained nuclei in each area by dividing the obtained volume by the average single nucleus volume for the area. To obtain the percentage of c-Fos positive cells, the number of c-Fos positive cells were divided by the total number of cells. For all other experiments, 40um sections were imaged using a single plane scan. To quantify c-Fos, CA1 segments were cropped to an area of 30,000um^2^ and imported to a grayscale image into FIJI. A cell count macro developed by Kyle Puhger was then used to process the image (https://github.com/kpuhger/imagej_cellcount). Briefly, the macro enhanced contrast of the image, removed outlier pixels (set by the user), then a gaussian blur is applied, and finally the maxima is identified. To quantify TH-expression, segments were cropped to an area of 30,000um^2^. After importing images to FIJI, images were cropped to an area of 30,000um^2^ for each segment of CA1. Images were then processed by using despeckle, removing outliers, and subtracting background. The mean intensity of the image was then calculated to quantify TH-expression.

### Statistics

No statistical methods were used to predetermine sample sizes. The sample sizes were chosen based on published studies and current standards in the field. This study’s design and its analysis were not preregistered. c-Fos and TH expression differences were analyzed with a two-way repeated measures ANOVA and post-hoc comparisons were made using Sidak’s multiple comparisons test. Activity levels during sample session and discrimination index were analyzed with a two-tailed unpaired t-test.

## Supporting information

S1 Fig Graphical abstract. (PDF)
