# Supplementary figures and images for: Object location memories recruit distal CA1 and catecholaminergic inputs to proximodistal CA1
Source: PLoS One. 2025 Dec 4;20(12):e0337834. doi: 10.1371/journal.pone.0337834 (PMC12677530; doi:10.1371/journal.pone.0337834)

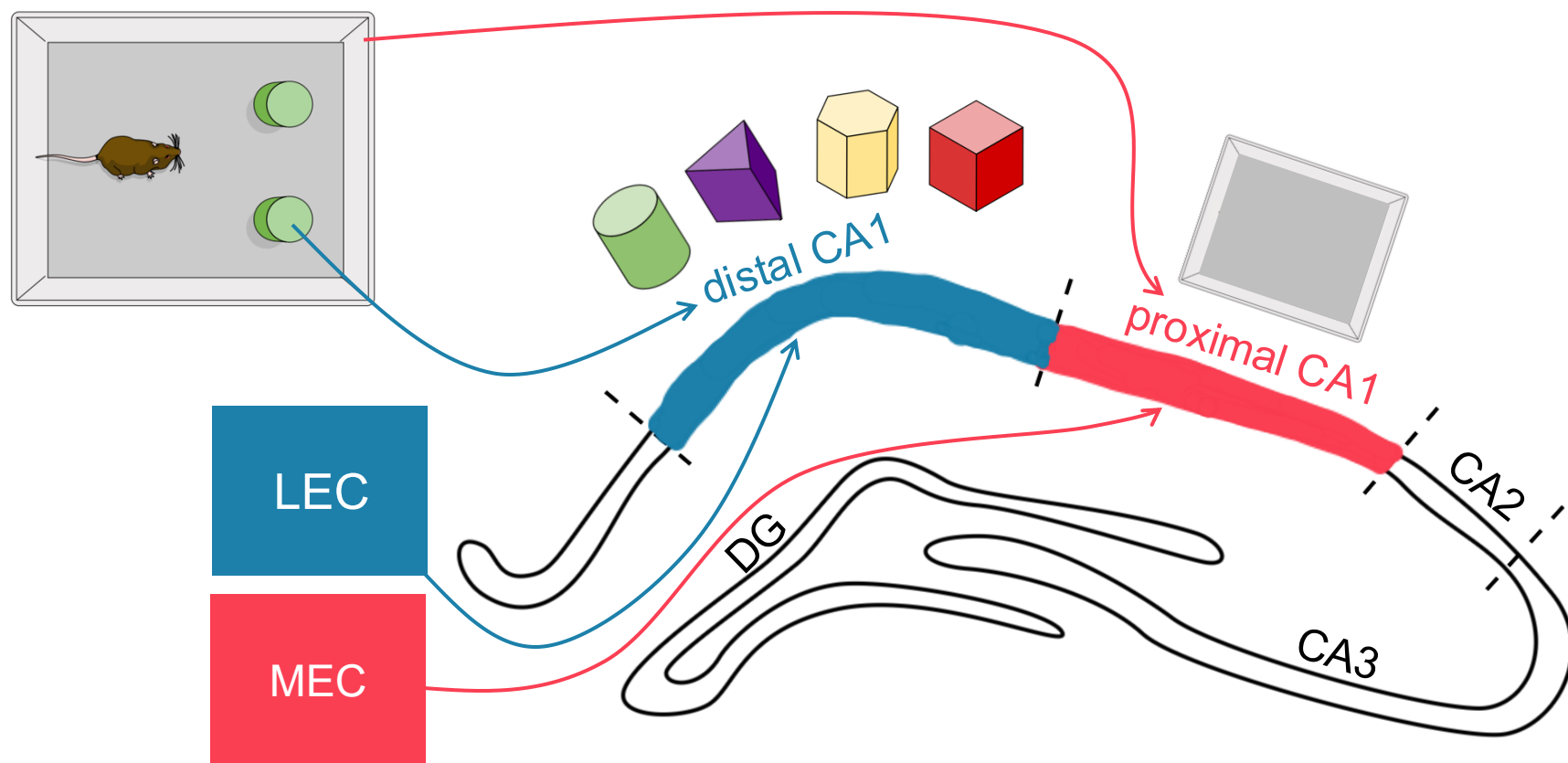

### Experiment 1:

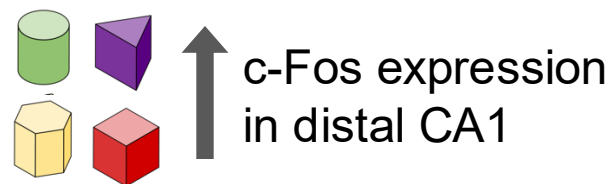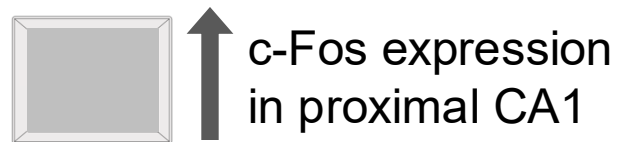

### Experiment 2 and 3:

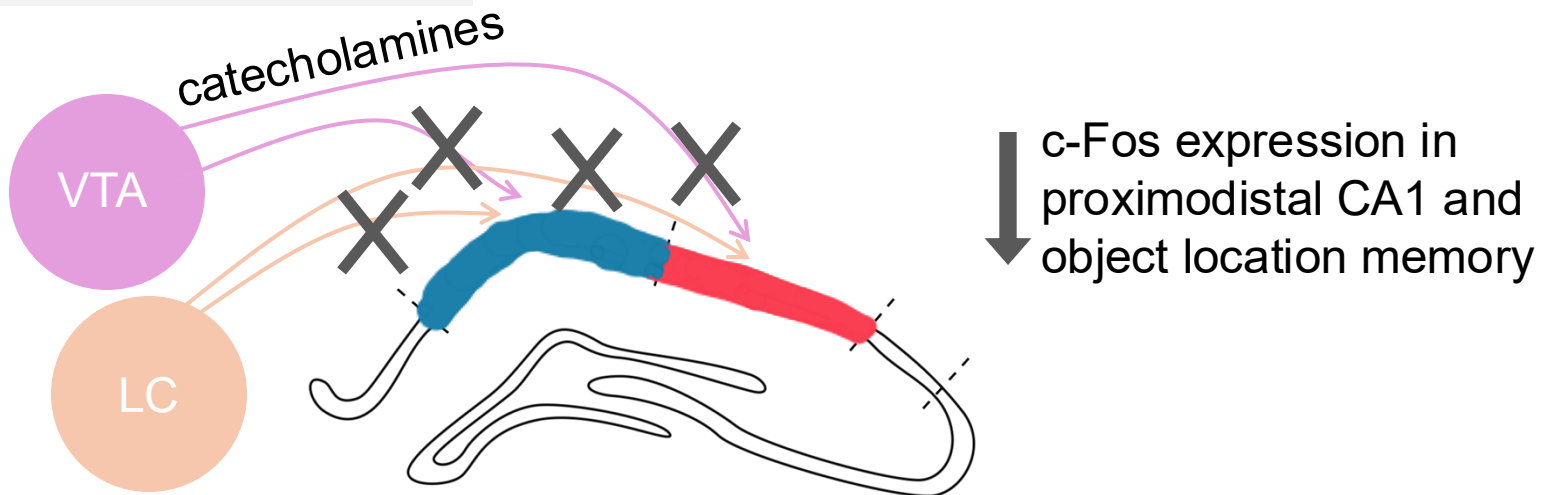

Supplement: S1 Fig — (PDF) [file pone.0337834.s001.pdf]
